# The Questionnaire of Intention to Help in VAW Cases (QIHVC): Development and preliminary results

**DOI:** 10.3389/fpsyg.2023.1153678

**Published:** 2023-03-22

**Authors:** Victoria A. Ferrer-Perez, Andrés Sánchez-Prada, Esperanza Bosch-Fiol, Carmen Delgado-Alvarez, Leila I. Vázquez-González, Ainara Nardi-Rodriguez

**Affiliations:** ^1^Faculty of Psychology, University of the Balearic Islands, Palma, Spain; ^2^Faculty of Psychology, Pontifical University of Salamanca, Salamanca, Spain; ^3^Faculty of Psychology, Miguel Hernández University of Elche, Elche, Spain

**Keywords:** violence against women, intimate partner violence, sexual harassment, street harassment, bystander response

## Abstract

**Introduction:**

Violence against women (VAW) is a worldwide social and health problem of epidemic proportions. This violence is preventable, and bystander programs are one of the possible preventative strategies. The main purpose of this research was to develop a tool that, by applying a contrastive methodology for its application in different forms of violence (forms of gender-based violence, such as intimate partner VAW, sexual harassment at work, and street harassment, and common violence, such as a robbery), would allow measuring the probability of occurrence of bystander response in the face of these types of violence with good evidence of content validity.

**Method:**

Firstly (Study 1), an initial version of a measure tool, the Questionnaire of Intention to Help in VAW Cases (QIHVC), was developed; secondly (Study 2), a Delphi (modified) study was carried out to obtain valid, content-based evidence; and finally (Study 3), a pilot study was carried out to evaluate the appropriate functioning of the QIHVC and, if required, to make any necessary adjustments.

**Results and discussion:**

The main result is the development of a set of case scenarios and a questionnaire related to its content which constitutes the QIHVC and, in its initial approximation, seems to constitute an adequate and sensible tool to capture the differences between the characterizations of common violence and VAW and in the possible response of bystanders in the face of such violence.

## Introduction

Violence against women (VAW) is a worldwide social and health problem of epidemic proportions (World Health Organization [WHO], 2018; Sardinha et al., 2022) and one of the most egregious and prevalent human rights violations against women and girls (UNWomen, 2015; Editorial, 2022). However, it is not an inevitable problem but rather is preventable (UNWomen-WHO, 2020). Different alternatives have been put forth as effective preventative strategies, including the development of individual skills to carry out preventative action, the creation of training groups to promote changes in social norms regarding masculinity or gender equality, or bystander programs (UNWomen, 2015). Within this context, this work focuses on studying bystander response and how it can be measured.

Within the scope of VAW, we refer to bystanders (or witnesses) as those individuals who are involved in offensive or violent acts but neither as victim nor as perpetrator (Banyard et al., 2005; Katz, 2006; Fenton et al., 2016; European Institute for Gender Equality [EIGE], 2020). That is, they are witness to the violence or to the conditions that perpetuate such acts, they are present immediately prior, during or after the incident, or they are aware of its occurrence and, despite having no direct participation in the incident, they do have the possibility to intervene by either helping the victim, perpetuating the violence or doing nothing at all (Banyard et al., 2005; Powell, 2014; Hamby et al., 2016).

Although some authors consider all individuals who form part of the community to be possible bystanders in cases of VAW (e.g., Waltermaurer, 2012), others make a distinction between bystanders, understood to be non-professional adults (including family members, friends, colleagues, neighbors, acquaintances, etc.) who observe, suspect or have knowledge of VAW, and professionals who work in any field related to such violence (including those who work in a judicial capacity, in health and social services, or in specialized services for victims of VAW, the police, etc.) (e.g., Herrero et al., 2017; European Institute for Gender Equality [EIGE], 2020). The present study focus on non-professional bystanders.

The field of crime prevention tends to distinguish passive bystanders, meaning those who know of or observe an incident but do nothing about it, and active bystanders (also referred to as prosocial bystanders), meaning those who engage in some type of bystander action, intervening in some capacity in response to an observed situation (Powell, 2014; Fenton et al., 2016). In fact, as recalled by Banyard (2015) and Rothman et al. (2019), the term bystander was originally used only to describe those who did nothing and, for that reason proposed speaking of “actionists” to refer to active bystanders who positively intervene in a violent situation.

The response of an “actionist” may contribute not only toward the prevention of VAW but also its consequences, and can manifest in different ways (McMahon and Banyard, 2012; Powell, 2014; Rothman et al., 2019; European Institute for Gender Equality [EIGE], 2020). Thus, for example, and using sexual violence as a reference, different bystander response opportunities have been classified as either reactive or proactive (McMahon and Banyard, 2012; Rothman et al., 2019): reactive responses would include actions that are carried our prior to, during or after a high-risk situation for the victim, including acts of primary prevention (such as informing somebody that their drink has been spiked), secondary prevention (such as calling the police or physically confronting the aggressor to prevent the aggression), or tertiary prevention (such as accompanying the victim to file a report or offering support); proactive responses would include actions that can be performed at any moment to modify social norms and attitudes that perpetuate violence and to promote healthy and non-violent relationships (such as participating in information or sensitivity campaigns). Other authors distinguish between intervention responses, which put an end to the violent situation, and responses focused on the prevention of violence, whether primary, secondary or tertiary prevention (e.g., Powell, 2014). In the case of intimate partner violence against women (IPVAW), the European Institute of Gender Equality (European Institute for Gender Equality [EIGE], 2020) distinguishes between mediation response (including lending help, support or understanding to the victim; helping the victim in choosing a course of action, intervening in a violent situation; or confronting the aggressor) y reporting responses (including informing or reporting a case of IPVAW to the police or authorities). In short, upon taking into account all of these possibilities, we can conclude that bystander response can be either positive and helpful if it contributes in any way toward halting VAW and/or protecting the victim, or non-helpful if it does not support the victim, remaining neutral, or somehow facilitating the occurrence of the VAW, and negative if it recriminates or blames the victim.

Findings have shown that a considerable number of cases of VAW occur in the presence of bystanders (Burn, 2009; Gracia et al., 2018; European Institute for Gender Equality [EIGE], 2020; León et al., 2022; Sánchez-Prada et al., 2022) and suggest the need and relevance for working with these individuals and convert them into “actionists,” active agents of prevention (McMahon and Banyard, 2012; Fenton and Mott, 2017; Fenton et al., 2019; McMahon et al., 2019; Rothman et al., 2019). This leads to the development of intervention programs to modify attitudes and beliefs held by bystanders (such as possible sexist attitudes or blaming the victim) and to strengthen their active responses (reactive and/or proactive) toward the victims (UNWomen, 2015). An important part of these programs, and the research on which they are based, has focused on cases of sexual VAW (Crooks et al., 2019; Kettrey and Marx, 2019; McMahon et al., 2019; Mainwaring et al., 2022; Woods et al., 2022), although they have been applied to others forms of VAW, such as IPVAW (Banyard et al., 2020; European Institute for Gender Equality [EIGE], 2020; Sprang et al., 2020).

A critical component for maximizing the efficacy and efficiency of these programs is to understand and analyze the bystander’s response (Bush et al., 2019) in addition to both their actual conduct and their willingness to act or intervene given that, as indicated in the Theory of Planned Behavior (Ajzen, 1985; Fishbein and Ajzen, 2010), intention constitutes a valuable predictor of conduct. Likewise, it is important to understand both the factors that facilitate active bystander response and the barriers that impede it (Burn, 2009; Gracia et al., 2018; European Institute for Gender Equality [EIGE], 2020; Álvarez-García et al., 2021; Mainwaring et al., 2022). Overall, there is a fundamental need to have the most valid assessment tools possible (Parrott et al., 2012; Banyard et al., 2014; McMahon et al., 2014, 2017, 2019; Jouriles et al., 2016; Bush et al., 2019).

One of the most commonly used techniques for measuring bystander responses are self-report questionnaires (Bush et al., 2019), focused both on bystander behavior, such as active participation in the incident, and the willingness to intervene (McMahon et al., 2014). Various questionnaires have been published in recent years, contributing significantly to this field of study.

As an example, the Bystander Behaviors Scale (BBS; Banyard et al., 2005, 2007, 2014; Banyard and Moynihan, 2011) originally included 51 items [later reduced to 20 in the Bystander Behavior Scale – Revised (BBS-R; McMahon et al., 2014)] which can be used to measure the helping behavior of bystanders with friends and strangers, whether in the context of sexual assault or abusive relationships, and to evaluate high-risk situations, access to resources, victim support and filing a complaint of physical violence, or proactive and safe behavior in open spaces (e.g., “Call the police or authorities if you saw a group of males bothering a female in a parking lot or similar situation”; or “Confront a friend if you heard rumors that they forced someone to have sex”). Participants in the survey should indicate whether they have engaged in this type of conduct in the previous 2 months, and the number of affirmative responses allows us to create an index for responsive bystander behavior (Jouriles et al., 2016). It should be said that, although it is a widely used questionnaire (i.e., Jouriles et al., 2020; Cascardi et al., 2021), it is not without limitations. One of the main constraints is that, although the meaning of the affirmative responses is clear (occurrence of conduct), the negative responses can be subject to various interpretations (e.g., that the incident did not take place, that when it took place the person did not recognize it as an opportunity to intervene, did nothing or did something different from what is described in the item) (Jouriles et al., 2016; McMahon et al., 2017). For this reason, some authors have proposed adding new response options relative to the number of times a response has occurred (e.g., Coker et al., 2011), or the lack of opportunity to engage (e.g., McMahon et al., 2017; Bush et al., 2019).

The Bystander Attitudes Scale (BAS; Banyard et al., 2005), later called Bystander Intention to Help Scale (Banyard and Moynihan, 2011), includes the same 51 items as the BBS [also reduced to 20 in the Bystander Attitudes Scale – Revised (BAS-R; McMahon et al., 2014)] used to evaluate the willingness to intervene in the face of high-risk situations, accompanying victims to file a report, reporting the violence to the police or authorities, and other proactive actions. For each item the participants use a 5-point scale to indicate (ranging from 1, not likely, to 5 very likely) the likelihood of engaging in the behavior described [e.g., “(How likely are you to) call the police or authorities if you saw a group of males bothering a female in a parking lot or similar situation?” or “(How likely are you to) confront a friend if you heard rumors that they forced someone to have sex?”]. In this case the answers can be tallied to obtain an index of willingness to intervene (Jouriles et al., 2016). As expected, and in accordance with the Theory of Planned Behavior (Ajzen, 1985; Fishbein and Ajzen, 2010), various studies (e.g., Banyard et al., 2007; Jouriles et al., 2016) have observed a significant and positive correlation between the willingness to intervene, as measured with the BBS, and bystander response, as measured by BAS.

The Bystander Intervention Behavior Scale (BIB; Burn, 2009) also evaluates willingness to intervene. More specifically it evaluates the differential probability between male and female adolescents of intervening in cases of sexual violence when the incident of violence occurs to a friend (4 items) or to a stranger (4 items). Participants must indicate on a 7-point scale (ranging from 1, completely disagree, to 7, completely agree) the extent to which they agree with the behavior described (e.g., “I remind my female friends to take actions to reduce sexual assault risk” or “If I see a man pressuring a woman to leave a party or bar with him, I intervene” on the scale used for females; and “I discourage my friends from talking about women in sexually degrading ways” or “I discourage strangers or acquaintances if I hear them talking about women in sexually degrading ways” on the scale used for males). The average score of the 8 items is used to create a bystander intervention index. These scales present indexes of adequate reliability, although they have not been used as often as the previous scales.

The Critically Conscious Bystander Scale (CCBS; Johnson et al., 2019) is another measure of self-report. It includes 43 items and measures four factors, including bystander response in the face of sexual harassment and sexual assault, activism, and increased awareness of these types of violence. The primary characteristic of this questionnaire is that beyond focusing on reactive response in the face of high-risk violent behavior, it focuses on the study of proactive behavior.

In contrast to previous scales, the Willingness to Intervene in Cases of Intimate Partner Violence (WI-IPVAW; Gracia et al., 2018) focuses on bystander response to IPVAW. This scale was developed in Spain and also indicates adequate psychometric properties. It includes 31 items that describe different situations of IPVAW in different contexts and the possible types of bystander response (e.g., “In a supermarket, if a man insulted his wife, I would ignore the situation”). Participants must indicate on a 6-point scale (ranging from 1, Not likely, to 6, Highly likely) the likelihood of intervening in the hypothetical scenarios. This questionnaire incorporates a bifactorial structure, including a general, non-specific factor relative to willingness to intervene in cases of IPVAW, which includes all items, and 3 specific factors relative to preferred forms of intervention (“call the police,” “personal involvement,” and “not my concern” factors, as defined by the authors). In addition to the complete version, there are two short versions of 9 and 5 items, respectively.

As previously noted, the different questionnaires mentioned in this study contain, overall, adequate psychometric properties and, beyond their internal consistency and their correlations with other variables related to theoretical presuppositions on the subject, have the advantage of being quick and easy to administer and calculate, and eliciting information involving different types of behavior in a wide range of situations (Jouriles et al., 2016). Nevertheless, due to their self-report nature (and especially if they are retrospective, as in several cases) they are not without constraints, including (Jouriles et al., 2016; McMahon et al., 2017): the effects of social desirability and the fact that people do not always respond accurately about what they said or did; the difficulties to correctly understand and interpret the characteristics of the situations about which they are asked (such as the seriousness, the danger involved, etc.); or the fact that the response options offered do not always reflect or adequately describe all of the available possibilities.

Precisely due to these limitations, other instruments have been developed to have the participants placed in the situation and offer a more direct measurement to the bystander responses. Thus, for example, to generate a situation involving exposure to sexual violence, Parrott et al. (2012) used film clips containing or not containing sexually explicit content, and Jouriles et al. (2016) used virtual reality to create a simulation. Other authors (e.g., Philpot et al., 2019) have even suggested analyzing bystander response in real-life violent or dangerous situations using CCTV (closed-circuit television) recordings.

However, this type of measurement is not without limitations either, including, among others, greater financial and human support than simply the use of self-reporting, technical or ethical problems, or the difficulty in representing or simulating certain VAW situations and the possible forms of bystander response (Jouriles et al., 2016).

Another variation of the previous study is the design of vignettes or case scenarios that present the participants with a particular situation after which they are asked questions. These types of tools have been widely used to study the perception of sexual assault (Papendick and Bohner, 2017), and also to study bystander response in these cases. To that end, for example, Katz et al. (2015), designed a scenario to describe a high-risk situation of sexual assault in a party, alternating the variable of knowing or not knowing the victim; Levy and Ben-David (2015) presented the description of a rape, alternating the variables of age and personal circumstances of the victim at the moment of the aggression; and Franklin and Garza (2021) described an intimate encounter between two students which ended in sexual assault, alternating the variables of the relationship (how well they knew each other) between the aggressor and the victim and the victim’s skin color. Other studies of bystander response in the case of IPVAW have used various scenarios. For example, Cinquegrana et al. (2018) presented a case of IPVAW alternating in the description the possible cause of violence; and León (2020) and León et al. (2022) presented a case of violence in which six variables were modified in the description: the situation that had led to the violence; the type of violence; the frequency of the occurrence; the origin of the couple involved; the adherence to traditional gender roles; and the possible cause of the violence.

Related to the present study, as previously noted, the methodology involving case scenarios is a useful contrast to the study involving bystander response in the case of sexual violence (e.g., Katz et al., 2015; Levy and Ben-David, 2015; Franklin and Garza, 2021) as well as IPVAW (e.g., Rincón-Neira, 2017; Cinquegrana et al., 2018; León et al., 2022). For this reason we decided to use this evaluation strategy in our research.

Specifically, our research focuses on the study of bystander response in the case of three forms of VAW, all of which are particularly important within the context of Spain, where our study takes place, given that: (a) IPVAW, which constitutes the most prevalent form of VAW on a global scale (World Health Organization [WHO], 2018; Sardinha et al., 2022) has been the object of focus in Spain both at a legislative level (including the development of the Organic Law 1/2004, 28 December, for Comprehensive Measures of Protection against Gender Violence), as well as at a social level and within research in general and on behalf of our research group; (b) Sexual Harassment in the Workplace (SHW), which, despite its lesser prevalence, has also been the object of legislative focus (Organic Law 3/2007, 22 March, for effective equality between women and men, and Organic Law 10/2022, 6 September, full guarantee of sexual freedom); and (c) street harassment (STH), understood as “the sexual harassment directed at women by a stranger in a public place” (Bowman, 1993, p. 51), specifying “undesired comments, gestures and actions forced upon an unknown person in a public space and without that person’s consent” (Plan Internacional, 2019), and which, according to preliminary research, constitutes an extraordinarily prevalent form of VAW especially among young women (Ferrer-Perez et al., 2021).

Given that, to the best of our knowledge, there are no existing tools to evaluate bystander response in the case of these three forms of VAW, the main purpose of this work is to present the development of a tool that applies a scenario-based methodology and makes it possible to measure the probability of the provided responses with good evidence of content validity. To this end, it is important to emphasize that, while measuring real bystander response is important, so too is evaluating one’s inclination to intervene (McMahon et al., 2015, 2019) given that intention constitutes one of the primary predictors of subsequent effective behavior (Ajzen, 1985; Fishbein and Ajzen, 2010), offers important keys to understand how to motivate individuals to participate as “actionists” (McMahon et al., 2015), and also constitutes one of the most frequent positive results after applying intervention programs with bystanders (Katz and Moore, 2013; McMahon et al., 2019). For these reasons, the decision was made to focus the measuring tool on the evaluation of intention or probability (in place of real response).

In an effort to reach this objective, a research survey was prepared, in the line of previous studied that have developed new procedures, tools, instruments, or tests to study psychometric properties (Montero and León, 2007; Ato et al., 2013). To begin (Study 1) an initial version of the Questionnaire of Intention to Help in VAW Cases (QIHVC) was developed; after that, (Study 2) a Delphi (modified) study was carried out to obtain valid, content-based evidence (American Educational Research Association [AERA] et al., 2014); and finally, (Study 3) a pilot study was carried out to evaluate the appropriate functioning of the tool and, if required, to make any necessary adjustments.

## Study 1: Develop the initial version of the Questionnaire of Intention to Help in VAW Cases

The aim of this first study was to develop a first version of the QIHVC.

### Materials and methods

#### Participants

Participants in this first study include individuals who form part of the research team and who have signed this work, along with junior collaborators who were involved in the project at that time, all of whom have training in VAW.

#### Procedure

Previous questionnaires for the evaluation of bystander response were used as a starting point in the design of the QIHVC (e.g., Banyard et al., 2005, 2007, 2014; Burn, 2009; Banyard and Moynihan, 2011; Coker et al., 2011; McMahon et al., 2014, 2017; Katz et al., 2015; Levy and Ben-David, 2015; Jouriles et al., 2016; Cinquegrana et al., 2018; Gracia et al., 2018; Bush et al., 2019; Johnson et al., 2019; León, 2020; Franklin and Garza, 2021; León et al., 2022), in particular those that had defined case-scenarios (e.g., Katz et al., 2015; Levy and Ben-David, 2015; Cinquegrana et al., 2018; León, 2020; León et al., 2022).

To begin, the specific forms of violence to be included were selected. As previously noted, three forms of VAW were chosen: IPVAW, SHW, and STH. Additionally, and as a means of control, a form of non-gender-based violence, specifically robbery, was also selected, with two differentiated variables according to whether the victim was male or female (RMV and RFV). Four junior collaborators all of whom are trained in VAW were then selected from the research team to prepare and agree on a description for each form of violence used as a case scenario, with the following conditions: (1) the situations described had to be as real and credible as possible; (2) the types of violence described had to be comparable in terms of the intensity of violence used by the aggressor, the presence of possible facilitating circumstances, the originator of the aggression and the possible risks and consequences for the bystanders; and (3) given the relevance of the bystander effect (see Darley and Latané, 1968, 1970; Latané and Darley, 1968), each type of violence required two scenarios, one with a single bystander (the participant) and another with several bystanders (the participant and other individuals). The first meeting was held with the collaborators to explain the objective and conditions that they had to meet in each case scenario. After that the collaborators worked with a partner and then in a group to share their ideas and reach an agreement for the final descriptions. After several rounds, and subsequent to combining the different levels of the relevant variables (type of violence and number of bystanders), the scenarios were defined (*n* = 10) and are described Table 1.

**TABLE 1:** Case scenarios designed.

		Number of bystanders	
		**Single bystander (the respondent)**	**Several bystanders**
Type of violence	Violence non gender-based: RMV	Case scenario 1	Case scenario 6
	Violence non gender-based: RFV	Case scenario 2	Case scenario 7
	VAW: IPVAW	Case scenario 3	Case scenario 8
	VAW: SHW	Case scenario 4	Case scenario 9
	VAW: STH	Case scenario 5	Case scenario 10

RMV, robbery – male victim; RFV, robbery – female victim; IPVAW, intimate partner violence against women; SHW, sexual harassment at work; STH, street harassment.

Finally, the member of the research team (who have signed this study) reviewed the vignettes, first individually and then as a group, until reaching a consensus on the wording with regard to the following aspects: (1) the situations described for each scenario had to be equally credible; (2) the situations were not to include physical violence (robbery due to carelessness in the case of RMV and RFV; psychological violence in the form of verbal aggression in the cases of IPVAW, SHW, and STH; (3) all situations presented had to include circumstantial aspects that could facilitate the activation of stereotypes for blaming the victim (e.g., leaving possessions within reach of others in the case of RMV and RFV; alcohol consumption and/or attire in the case of de IPVAW, SHW and STH); (4) the description of the scenarios could leave no doubt as to the originator of the aggression; and (5) in all case scenarios, the proposed intervention would assume the same level of risk to the bystander.

### Results

The developed case scenarios, which include a description of three situations of VAW (corresponding to IPVAW, SHW, and STH) and one common form of violence with two variables according to the gender of the victim (RMV and RFV), are presented in Supplementary Appendix 1.

Next, and referencing the questions included in previous studies (e.g., León, 2020; León et al., 2022) a questionnaire was developed with similar wording used for each of the scenarios and related to the following variables (see Supplementary Appendix 2):

– Perceived seriousness of the violence described in each scenario, understood as the importance attributed to the harm or danger for the victim, measured on a Likert scale ranging from 1, Not serious, to 4, Very serious.
– Responsibility attributed to the victim, related to the victims’ degree of responsibility in the violence, measured on a Likert scale ranging from 1, Not responsible, to 4, Very responsible.
– Responsibility attributed to the perpetrator, related to the perpetrator’s degree of responsibility in the violence, measured on a Likert scale ranging from 1, Not responsible, to 4, Very responsible.
– Responsibility attributed to the bystander in acting as an active agent, that is, the extent to which the participant would consider him or herself responsible for intervening, measured on a Likert scale ranging from 1, Not responsible, to 4, Very responsible.
– Willingness to intervene: using several previous studies as a reference (e.g., Fundación FEDE. Social Research Service, 2012; León, 2020; León et al., 2022), addresses the intention or probability of carrying out a series of bystander responses. Specifically, 10 possible answers, either positive and helpful to, or negative and not helpful, and both active and passive (confronting the victim; confronting the aggressor; calling the police and confronting the aggressor; calling the police but not confronting the aggressor; trying to mediate between the victim and the aggressor; trying to help the victim; asking other people or witnesses for help; not knowing what to do; probably do nothing; and do nothing because “it’s not my concern”). Responses were given according to a 4 point Likert scale ranging 1, Not likely, to 4, Very likely).

### Discussion

Subsequent to the process described, an evaluation tool was obtained and considered adequate to reach the intended objectives (see Supplementary Appendix 1, 2). The proposal gathered the suggestions from previous studies on the topic (Banyard et al., 2005, 2007, 2014; Burn, 2009; Banyard and Moynihan, 2011; Coker et al., 2011; McMahon et al., 2014, 2017; Katz et al., 2015; Levy and Ben-David, 2015; Jouriles et al., 2016; Cinquegrana et al., 2018; Gracia et al., 2018; Bush et al., 2019; Johnson et al., 2019; León, 2020; Franklin and Garza, 2021; León et al., 2022) and incorporated a new element with the focus on three forms of VAW and one type of non-gender based violence as the control element.

With the aim of confirming its suitability, the following two studies were carried out.

## Study 2: Panel of experts (modified Delphi)

The aim of this second study was to determine the suitability of the case scenarios and the design of the corresponding questionnaires by subjecting them to an expert review.

### Materials and methods

#### Participants

Following the considerations of Pedrosa et al. (2013), thirty Spanish experts in VAW were invited to take part in a modified Delphi Study, of which 20 agreed to participate. Of these, 18 completed the entire procedure, 14 females and 4 males. All have a Ph.D. degree and research experience in one or more forms of VAW (16 in IPVAW; 3 in STH; and 2 in sexual violence). One is a practitioner specialized in gender-based violence, while 17 came from academic settings (16 from research groups from Behavioral Science Departments and 1 from a Law Department).

#### Instruments

Two templates were designed. Both contained the aim of the study, the instructions to follow by the experts and the definition of each one of the dimensions on which they had to base the assessment of the case scenarios and the items of the questionnaire.

The first template included the description of the different VAW scenarios designed in Study 1 (see Table 1 and Supplementary Appendix 1).

Experts had to answer to the following questions: “What type of violence is described in the scenario?” (pertinence dimension), “To what degree do you consider that this scenario is characteristic of the condition it is supposed to describe?” (representativeness dimension), “To what extent do you think that this scenario can give rise to more than one interpretation?” (ambiguity dimension), “To what extent do you think the scenario is easy for the reader to understand?” (comprehension dimension), and “To what extent do you consider the description of the setting to be clear and concise?” (clarity dimension).

Except for the pertinence dimension, experts had to mark with an x for each scenario if it described an RMV, RFV, IPVAW, SHW, or STH situation. For the remaining dimensions, experts had to answer with a 5-point Likert scale.

In the second template, experts were given a questionnaire related to the scenarios that contained questions related to the perceived seriousness of violence, the responsibility attributed to the victim, to the perpetrator, and to the bystander, and the willingness to intervene by engaging in different bystander responses (see Supplementary Appendix 2). They used a 5 point Liker scale for their assessment of each question with regard to: (1) its pertinence (if the question is pertinent with regard to the scenario); (2) its representativeness (the degree to which the question is accurate to measure the perceived seriousness of the scenario, the responsibility attributed to victims, aggressors, and bystander’s responsibility in acting and performing helping behaviors); (3) its comprehension (to what extent the question is easy to understand); and (4) its clarity (to what extent the question is clear and concise).

At the end of both templates, experts had the opportunity to provide any commentary they believed relevant to improve the description of the vignettes and the items in the questionnaire items.

#### Procedure

To gather the sample, the research group first completed the Knowledge Resource Nomination Worksheet (KRNW) (Okoli and Pawlowski, 2004) which listed all the participating well-known Spanish experts and included the following criteria: working in an academic or clinical context, have a Ph.D., and research experience in any of the different forms of VAW under study. Next, the authors contacted the experts via email to invite them to participate in the study, explaining the aim of the project and what their participation would consist of. Each expert participant received an email with the two templates (previously described) as attachments and was asked to confirm the email reception and their participation in a one round study, that is, the modified Delphi study (Lentz, 2007).

After receiving the response from each of the experts, we performed a descriptive analysis of the different dimensions assessed in both templates [mean and standard deviation (SD)] and a qualitative analysis of the experts’ commentaries. Based on these results, we discussed and made decisions about the final format of the scenarios and questionnaire.

To establish the criteria of consensus among experts, we first reviewed the literature to find the different criteria used in Delphi studies. To this respect, we must point out that researchers are generally the ones who establish the criterion, normally based on a descriptive analysis parameter such as the median or the mode, or the mean and SD (García-Valdés and Suárez-Marín, 2013). In this study, to ensure there was a consensus among experts regarding the adequacy of the scenarios and the questions assessed, we decided to use two criteria to obtain the best content validity: (1) at least 13 out of the 18 experts had to agree on the assessment of the dimensions. This criterion was pulled out from Ayre and Scally (2013) Content Validity Ratio table (CVR). Based on exact binomial probabilities, the CVR table indicates the minimum number of experts that had to agree on an item according to the total number of experts that participated, so that agreement is not just by chance. Missing responses did not affect this criterion; and (2) at least 13 experts had to agree by giving the highest score to the dimensions measured.

### Results

#### The case scenarios

The results of the experts’ assessment of each scenario can be found in Table 2.

**TABLE 2:** Experts’ assessment of the case scenarios.

Scenarios	Pertinence	Representativeness		Ambiguity		Comprehension		Clarity	
	**%**	***n***	***M* (SE)**	***n***	***M* (SE)**	***n***	***M*** **(SE)**	***n***	***M* (SE)**
RMV (single witness)	94.5	10	3.89 (1.45)	8	4.22 (0.88)	15	4.83 (0.38)	15	4.83 (0.38)
RMV (several witnesses)	94.5	10	3.89 (1.45)	8	4.22 (0.88)	14	4.78 (0.43)	15	4.83 (0.38)
RFV (single witness)	94.5	10	3.89 (1.45)	8	4.22 (0.88)	14	4.78 (0.43)	15	4.83 (0.38)
RFV (several witnesses)	94.5	10	3.89 (1.45)	8	4.17 (0.92)	14	4.78 (0.43)	13	4.83 (0.38)
IPVAW (single witness)	100	16	4.78 (0.73)	10	4.44 (0.70)	15	4.83 (0.38)	14	4.72 (0.57)
IPVAW (several witnesses)	100	16	4.78 (0.73)	12	4.61 (0.61)	16	4.89 (0.32)	16	4.83 (0.51)
SHW (single witness)	94.5	13	4.65 (0.70)	14	4.76 (0.56)	17	5.00 (0.00)	16	5.00 (0.00)
SHW (several witnesses)	100	14	4.67 (0.68)	15	4.78 (0.55)	18	5.00 (0.00)	18	5.00 (0.00)
STH (single witness)	100	16	4.83 (0.51)	14	4.78 (0.43)	18	5.00 (0.00)	16	4.89 (0.32)
STH (several witnesses)	100	16	4.83 (0.51)	14	4.78 (0.43)	18	5.00 (0.00)	16	4.89 (0.32)

RMV, robbery – male victim; RFV, robbery – female victim; IPVAW, intimate partner violence against women; SHW, sexual harassment at work; STH, street harassment. *M*, estimated mean; SE, standard error.

##### Pertinence

All the experts correctly related each one of the scenarios to its corresponding condition, except for one expert who considered that the four situations describing a robbery technically reflected a case of theft. One expert did not answer with regard to the SHW condition with a single bystander (missing).

##### Representativeness

Between 13 and 16 experts considered that the scenarios were highly representative of the different forms of VAW, attributing the maximum score to the scenarios in this dimension. By contrast, only 10 were in agreement with regard to the four situations of robbery. The main problem was the technical difference between robbery and theft. Another point was that the robbery scenes took place in an empty bar, so it was considered quite unlikely that a person (the victim) would leave their belongings on the bar.

##### Ambiguity

The robbery and IPVAW scenarios did not reach the consensus criteria required (*n* ≥ 13) to conclude that the scenarios were not ambiguous. To reduce the ambiguity, experts recommended emphasizing the fact that the witness/es were observing the robbery. Although the SHW scenario did reach a consensus regarding the lack of ambiguity, one expert considered that some people may not interpret the touching of a victim’s knee to be a behavior with sexual intentions. Another expert points out that it is the only scenario where the victim asked for help with her eyes, and recommended erasing this nuance to equate the scenarios. Regarding the STH situation, one expert suggested explicitly stating that the victim and aggressor did not know each other.

##### Comprehension and clarity

All the scenarios reached the minimum number of experts required to consider that they were easy to understand (*n* = 14–18), clear and concise (*n* = 13–18). However, seven experts commented that in the IPVAW scenario, the sentence “once they started living together things changed” was a bit confusing since it was not known if things changed for better or worse, even if it could be deduced later on. Two more experts considered it strange that only one neighbor (the reader in the one single bystander scenario) would hear the episodes of violence in a building.

#### The questionnaire

At least, 13 to 18 experts attributed the highest scores in pertinence, representativeness, comprehension, and clarity of the questions to the perceived seriousness of the scenario, and the responsibility of the victim, aggressor and bystander in the described case scenarios (Table 3).

**TABLE 3:** Experts’ assessment of the items related to the perceived seriousness and agents’ responsibility in each case scenario.

	Pertinence		Representativeness		Comprehension		Clarity	
**Case scenarios**	***n***	***M* (SE)**	***n***	***M* (SE)**	***n***	***M* (SE)**	***n***	***M* (SE)**
**Perceived seriousness**								
RMV (single/several witnesses)	14	4.72 (0.57)	14	4.72 (0.57)	15	4.72 (0.57)	17	4.94 (0.23)
RFV (single/several witnesses)	14	4.78 (0.43)	14	4.78 (0.43)	15	4.72 (0.67)	17	5.00 (0.00)
IPVAW (single/several witnesses)	16	4.94 (0.24)	16	4.94 (0.24)	16	4.88 (0.48)	17	4.89 (0.47)
SHW (single/several witnesses)	17	4.94 (0.24)	17	4.94 (0.24)	17	4.89 (0.47)	18	5.00 (0.00)
STH (single/several witnesses)	17	4.94 (0.24)	17	4.94 (0.24)	17	4.89 (0.47)	18	5.00 (0.00)
**Victim’s responsibility**								
RMV (single/several witnesses)	15	4.78 (0.55)	17	4.94 (0.23)	17	4.94 (0.23)	17	4.94 (0.23)
RFV (single/several witnesses)	15	4.78 (0.55)	17	4.94 (0.27)	17	4.94 (0.27)	17	4.94 (0.27)
IPVAW (single/several witnesses)	16	4.83 (0.51)	16	4.83 (0.51)	16	4.83 (0.51)	17	4.83 (0.51)
SHW (single/several witnesses)	16	4.78 (0.73)	16	4.78 (0.73)	16	4.78 (0.73)	16	4.78 (0.73)
STH (single/several witnesses)	16	4.83 (0.51)	16	4.83 (0.51)	16	4.83 (0.51)	16	4.83 (0.51)
**Aggressor’s responsibility**								
RMV (single/several witnesses)	17	4.78 (0.55)	17	4.94 (0.23)	17	4.94 (0.23)	18	4.94 (0.23)
RFV (single/several witnesses)	17	4.94 (0.24)	17	5.00 (0.00)	17	5.00 (0.00)	18	5.00 (0.00)
IPVAW (single/several witnesses)	17	5.00 (0.00)	17	5.00 (0.00)	17	5.00 (0.00)	17	5.00 (0.00)
SHW (single/several witnesses)	17	5.00 (0.00)	17	5.00 (0.00)	17	5.00 (0.00)	17	5.00 (0.00)
STH (single/several witnesses)	17	5.00 (0.00)	17	5.00 (0.00)	17	5.00 (0.00)	17	5.00 (0.00)
**Bystander’s responsibility**								
RMV (single/several witnesses)	15	4.83 (0.38)	16	4.83 (0.51)	13	4.56 (0.78)	13	4.61 (0.70)
RFV (single/several witnesses)	15	4.83 (0.40)	16	4.83 (0.51)	14	4.61 (0.78)	15	4.72 (0.67)
IPVAW (single/several witnesses)	16	4.94 (0.24)	16	4.83 (0.51)	16	4.72 (0.67)	17	4.72 (0.67)
SHW (single/several witnesses)	16	4.94 (0.24)	16	4.83 (0.51)	16	4.72 (0.67)	16	4.72 (0.67)
STH (single/several witnesses)	17	4.94 (0.24)	16	4.83 (0.51)	15	4.72 (0.67)	15	4.72 (0.67)

RMV, robbery – male victim; RFV, robbery – female victim; IPVAW, intimate partner violence against women; SHW, sexual harassment at work; STH, street harassment. *M*, estimated mean; SE, standard error.

However, three experts considered that the question “to what extent do you think that the victim caused the situation?” was strange because of the verb to cause, and one expert suggested changing it for to be responsible for the situation as it was formulated in the questions regarding the responsibility of the aggressor and witness.

The experts’ assessment of the questions related to the bystander’s behaviors in each case scenario is presented in Table 4.

**TABLE 4:** Experts’ assessment of the questions related to the helping and non-behaviors in each scenario.

Scenarios	Pertinence		Representativeness		Comprehension		Clarity	
	***n***	***M* (SE)**	***n***	***M* (SE)**	***n***	***M* (SE)**	***n***	***M* (SE)**
**Bystander’s behavior 1: I would confront the victim**								
RMV (single/several witnesses)	6	3.06 (1.70)	6	3.18 (1.66)	8	3.06 (1.70)	8	3.06 (1.70)
RFV (single/several witnesses)	7	3.24 (1.64)	7	3.41 (1.62)	9	4.06 (1.30)	10	4.18 (1.3)
IPVAW (single/several witnesses)	9	3.50 (1.70)	9	3.56 (1.65)	10	4.06 (1.30)	9	4.00 (1.30)
SHW (single/several witnesses)	9	3.56 (1.68)	9	3.56 (1.68)	11	4.11 (0.67)	11	4.11 (1.37)
STH (single/several witnesses)	10	3.72 (1.64)	10	3.78 (1.63)	12	4.33 (1.20)	12	4.33 (1.20)
**Bystander’s behavior 2: I would confront the perpetrator**								
RMV (single/several witnesses)	16	4.88 (0.48)	16	4.94 (0.24)	14	4.76 (0.56)	15	4.82 (0.53)
RFV (single/several witnesses)	16	4.88 (0.48)	16	4.94 (0.24)	13	4.65 (0.70)	14	4.71 (0.68)
IPVAW (single/several witnesses)	17	4.94 (0.24)	17	4.94 (0.24)	16	4.83 (0.51)	16	4.89 (0.32)
SHW (single/several witnesses)	16	4.72 (0.96)	17	4.94 (0.24)	16	4.83 (0.51)	17	4.94 (0.24)
STH (single/several witnesses)	17	4.89 (0.47)	17	4.83 (0.70)	16	4.83 (0.51)	17	4.94 (0.24)
**Bystander’s behavior 3: I would call the police and i would confront the perpetrator**								
RMV (single/several witnesses)	15	4.72 (0.75)	16	4.78 (0.73)	13	4.39 (1.19)	14	4.50 (1.15)
RFV (single/several witnesses)	13	4.50 (0.92	14	4.50 (1.04)	12	4.33 (1.18)	13	4.44 (1.15)
IPVAW (single/several witnesses)	16	4.78 (0.73)	16	4.78 (0.73)	14	4.44 (1.20)	13	4.44 (1.15)
SHW (single/several witnesses)	13	4.22 (1.40)	14	4.50 (1.10)	13	4.39 (0.14)	14	4.94 (1.10)
STH (single/several witnesses)	15	4.56 (1.15)	15	4.56 (1.15)	14	4.44 (1.20)	15	4.56 (1.15)
**Bystander’s behavior 4: I would call the police, but not confront the perpetrator**								
RMV (single/several witnesses)	15	4.78 (0.55)	16	4.83 (0.51)	13	4.44 (1.10)	14	4.56 (1.04)
RFV (single/several witnesses)	15	4.72 (0.75)	16	4.78 (0.73)	13	4.39 (1.19)	14	4.50 (1.15)
IPVAW (single/several witnesses)	16	4.78 (0.73)	16	4.78 (0.73)	14	4.50 (1.15)	14	4.50 (1.15)
SHW (single/several witnesses)	12	4.24 (1.30)	13	4.33 (1.24)	12	4.33 (1.14)	13	4.44 (1.10)
STH (single/several witnesses)	15	4.56 (1.15)	15	4.56 (1.15)	14	4.44 (1.20)	15	4.56 (1.15)
**Bystander’s behavior 5: I would try to mediate, if possible, between the victim and the perpetrator**								
RMV (single/several witnesses)	5	3.18 (1.55)	7	3.39 (1.54)	8	3.67 (1.45)	7	3.61 (1.42)
RFV (single/several witnesses)	7	3.35 (1.62)	9	3.76 (1.56)	9	4 (1.32)	9	3.88 (1.36)
IPVAW (single/several witnesses)	16	4.72 (0.96)	18	5	17	4.94 (0.24)	16	4.83 (0.51)
SHW (single/several witnesses)	12	4.12 (1.60)	14	4.39 (1.33)	14	4.56 (1.04)	13	4.50 (1.10)
STH (single/several witnesses)	14	4.22 (1.51)	15	4.44 (1.30)	14	4.44 (1.20)	13	4.33 (1.24)
**Bystander’s behavior 6: I would try to help the victim**								
RMV (single/several witnesses)	12	4.41 (1.00)	13	4.63 (0.88)	12	4.47 (0.94)	12	4.41 (1.00)
RFV (single/several witnesses)	15	4.61 (0.91)	15	4.56 (1.04)	14	4.56 (0.92)	14	4.56 (0.92)
IPVAW (single/several witnesses)	17	5.00 (0.00)	17	5.00 (0.00)	16	4.94 (0.24)	16	4.94 (0.24)
SHW (single/several witnesses)	17	5.00 (0.00)	17	5.00 (0.00)	16	4.94 (0.24)	16	4.94 (0.24)
STH (single/several witnesses)	17	5.00 (0.00)	17	5.00 (0.00)	16	4.94 (0.24)	16	4.94 (0.24)
**Bystander’s behavior 7: I would ask someone for help**								
RMV (single/several witnesses)	15	4.82 (0.53)	17	4.89 (0.47)	15	4.78 (0.55)	16	4.83 (0.51)
RFV (single/several witnesses)	17	4.89 (0.47)	17	4.89 (0.47)	16	4.83 (0.51)	16	4.83 (0.51)
IPVAW (single/several witnesses)	17	4.89 (0.47)	17	4.89 (0.47)	16	4.83 (0.51)	16	4.83 (0.51)
SHW (single/several witnesses)	17	4.89 (0.47)	17	4.89 (0.47)	16	4.83 (0.51)	16	4.83 (0.51)
STH (single/several witnesses)	17	4.89 (0.47)	17	4.89 (0.47)	16	4.83 (0.51)	16	4.83 (0.51)
**Bystander’s behavior 8: I would not know what to do**								
RMV (single/several witnesses)	18	5.00 (0.00)	17	4.94 (0.24)	17	4.89 (0.47)	17	4.89 (0.47)
RFV (single/several witnesses)	18	5.00 (0.00)	18	5.00 (0.00)	17	4.89 (0.47)	17	4.89 (0.47)
IPVAW (single/several witnesses)	18	5.00 (0.00)	18	5.00 (0.00)	17	4.89 (0.47)	17	4.89 (0.47)
SHW (single/several witnesses)	18	5.00 (0.00)	18	5.00 (0.00)	17	4.89 (0.47)	17	4.89 (0.47)
STH (single/several witnesses)	18	5.00 (0.00)	18	5.00 (0.00)	17	4.89 (0.47)	17	4.89 (0.47)
**Bystander’s behavior 9: I would probably do nothing**								
RMV (single/several witnesses)	16	4.76 (0.97)	16	4.76 (0.97)	16	4.88 (0.48)	16	4.88 (0.48)
RFV (single/several witnesses)	15	4.65 (1.06)	15	4.65 (1.06)	16	4.88 (0.48)	16	4.88 (0.48)
IPVAW (single/several witnesses)	14	4.73 (1.03)	14	4.59 (1.06)	14	4.81 (0.54)	15	4.88 (0.50)
SHW (single/several witnesses)	15	4.75 (1.00)	15	4.75 (1.00)	15	4.88 (0.50)	15	4.88 (0.50)
STH (single/several witnesses)	15	4.75 (1.00)	15	4.65 (1.06)	15	4.88 (0.50)	15	4.88 (0.50)
**Bystander’s behavior 10: I would do nothing (it is not my concern)**								
RMV (single/several witnesses)	16	4.67 (1.03)	16	4.67 (1.03)	16	4.83 (0.51)	16	4.72 (0.83)
RFV (single/several witnesses)	16	4.67 (1.03)	15	4.61 (1.03)	16	4.83 (0.51)	16	4.83 (0.51)
IPVAW (single/several witnesses)	16	4.67 (1.03)	16	4.67 (1.03)	17	4.89 (0.47)	17	4.89 (0.47)
SHW (single/several witnesses)	16	4.67 (1.03)	16	4.67 (1.03)	16	4.83 (0.51)	16	4.83 (0.51)
STH (single/several witnesses)	16	4.67 (1.03)	16	4.67 (1.03)	16	4.83 (0.51)	16	4.83 (0.51)

RMV, robbery – male victim; RFV, robbery – female victim; IPVAW, intimate partner violence against women; SHW, sexual harassment at work; STH, street harassment. *M*, estimated mean; SE, standard error.

As seen in the Table 4, the bystander’s behavior “to confront the victim” did not reach the minimum consensus (*n* ≥ 13) in pertinence, representativeness, comprehension, and clarity for any of the scenarios. The main reasons were that confronting the victim was labeled as a disturbing and implausible behavior, and experts considered mediation in the robbery scenes to be unlikely since the thief runs away. The verb “confront” was considered unclear since it could imply reacting or not reacting aggressively.

The bystander’s behavior “to call the police and confront the perpetrator” did not reach the minimum consensus in comprehension for the RFV scenario (*n* = 12), but it did for the rest of the robbery scenes. One expert pointed out that the verb “to confront” was unclear.

Neither the bystander’s behavior “to call the police but not confront the perpetrator” did not reach the minimum consensus among experts regarding its pertinence (*n* = 12) and comprehension (*n* = 12) in the SHW scenario. Two experts mentioned that calling the police would be an unusual behavior in a work context and that this issue could be extrapolated to the previous helping behavior. Three experts also recommended separating “calling the police” as a separate option, since a previous item already tackled the helping behavior “confronting the aggressor.”

Nor bystander’s behavior “mediating between victim and aggressor” did not reach the minimum consensus (*n* < 13) in pertinence, representativeness, comprehension, and clarity for any of the robbery scenarios (independently of the gender of the victims).

The helping behavior “try to help the victim” did not reach the minimum consensus (*n* = 12) among experts regarding its pertinence, comprehension, and clarity in RMV. The main reason pointed out by three experts was related to the verb “to help.” The equivalent used in Spanish had the connotation of “saving” the victim and since in the robbery scenarios the victims were not in a dangerous situation, experts considered it best to replace it with another verb that could serve to assess this helping behavior in all scenes.

In the rest of the bystander responses analyzed, the experts’ consensus reached the minimum required for each scenario on their pertinence, representativeness, comprehension, and clarity, ranging from 13 to 18 experts who assessed the above mentioned dimensions with the maximum score. However, it could be pointed that eight experts considered that the differences between the non-helping passive behaviors used [that is “I wouldn’t know what to do,” “I would probably do nothing,” and “I would do nothing (it’s not my concern)”] should be clearer by clarifying the reasons why a person would not know how to react and would do nothing in the two first questions.

### Discussion

In general terms, the results obtained in Delphi study point out that the scenarios and questionnaires showed good validity evidence based on the content, requiring few changes. In any case, and in order to improve the scenarios and questionnaire as much as possible, we discussed these results, taking into account all the experts’ observations, even on items that had reached the consensus criterion.

In this sense, to decrease the ambiguity of the robbery scenarios, and following the experts’ recommendations, we decided to restate more explicitly that the witness observed how the thief stole the mobile phone. However, although experts suggested improving the representativeness of these scenarios by not describing an empty bar and referring to them as theft scenarios, we decided not to do so. Respectively, the reason was that one of the variables being studied is the effect on helping behaviors for single bystander, and that the Spanish population usually refers to these type of scenarios as robbery (and not as theft, a more technical term). In contrast we adapted the robbery scenario to experts’ request to make the scenario more plausible in that a victim would leave the mobile on top of a table in a bar that he/she would frequent or know.

Addressing the suggestions made on the dimensions that reached consensus in the different scenarios in order to improve them, we decided to make the SHW behavior more evident by pointing that the boss was touching the victim’s thigh. We also decided not to mention that the victim asked for help with her eyes. In the STH situation we detailed that the victim and aggressor did not know each other. And in the IPVAW scenario, the clarity of the scene description was improved by shortening the sentences, deleting some sentences considered confusing by some experts, and by specifying that because of the floorplan of the houses in the building, either only one witness (single bystander condition) was able to hear the violent episodes, or all the neighbors could hear it (several witnesses condition).

Regarding the questionnaire, some items were modified to improve their pertinence, representativeness, comprehension, and clarity, based on the experts’ recommendations. For instance, the item “to mediate between victim and aggressor” was erased, “to confront the victim” was replaced by “to reproach the victim,” the item to “call the police” would be on its own (“without combining it with confronting the aggressor” or “not confronting the aggressor”), and this same item was nuanced with the option of “calling the authorities” to improve its pertinence to the SHW scenario. The question aiming to assess the victims’ responsibility for the situation was adapted, as suggested by experts, avoiding the verb “to cause.” Finally, we agreed with experts in specifying the reasons for not helping a victim as follows: “I would not know what to do, I would be mentally blocked,” “I would do nothing because it’s none of my concern,” and “I would do nothing for fear,” and we corrected this options in the specified sense. Thus, the 10 bystander responses initially included were finally reduced to 8: 4 positive or active bystander helping responses (“confront the perpetrator,” “call the police / alert the authorities,” “help the victim,” and “ask other people for help”), and 4 negative or passive bystander responses (“reproach the victim for her/his actions,” “do not know what to do, would freeze up,” “do nothing because it’s not my concern,” “do nothing out of fear”). All changes made to the QIHVC (scenario descriptions and questionnaire questions) can be found in Supplementary Appendix 2.

This study has some limitations. First of all, the great majority of experts come from the same discipline (Psychology) which could affect content validity by responding in a similar manner (Sireci and Faulkner-Bond, 2014). However, bystander behaviors and the bystander effect has been a field of study of great interest to Psychology, which has studied both topics in depth (Darley and Latané, 1968, 1970; Latané and Darley, 1968). Secondly, although 30 experts were invited, only 18 participated. Nevertheless, the number of experts participating in our study nearly doubled the recommendation in the literature for a content validity study (at least 10 subject matter experts) (Sireci and Faulkner-Bond, 2014). Finally, the length of the questionnaires could affect the experts’ responses. To mitigate this effect, experts had 1 month to return their assessments.

Regarding the strengths of this study, a type of quality control procedure that supports content validity consists of subjecting test items to the knowledge of experts to ensure their technical accuracy (Sireci and Faulkner-Bond, 2014). In addition, the criteria of consensus were strict (at least 13 out of 18 experts had to agree on a dimension by giving the maximum score) and furthermore, the experts’ consensus regarding scenarios and the adequacy of the items was high. Moreover, the authors incorporated the suggestions they believed would improve the scenarios and questionnaire even if the dimensions achieved consensus.

## Study 3: Pilot study

The aim of the third study was to explore the sensitivity of QIHVC to grasp the differences between the characterization of common violence and VAW in the possible bystander responses to these types of violence.

### Materials and methods

#### Participants

A convenience sample of 115 students from two Spanish universities took part in this study, 89 women (77.4%) and 25 men (21.7%). The average age was 21.37 years (SD = 2.79), ranging from 18 to 44 and with no significant differences between women and men [*t*(112) = 0.081, *p* = 0.936].

#### Instruments

The information was gathered by means of a questionnaire which included sociodemographic (gender and age) and the QIHVC, in its modified format, following the recommendations made in Study 2, as previously presented.

#### Procedure

A non-probabilistic sample of convenience was used. The data were gathered online using the Lime Survey platform. Specifically, students were provided with a link to the webpage where the questionnaire could be found. Upon initiating the survey a text appeared explaining the aims of the study, and access to the questionnaire answer sheet indicated consent to participate in the study. This was followed by questions used to gather sociodemographic data and, finally, the case scenarios of violence. It is important to note that the different case scenarios were presented in random order for each participant (using the randomization feature used in the Lime Survey platform).

#### Data analysis

A 5 × 2 quasi-experimental design was used with a within-subjects factor relative to the type of violence (RMV vs. RFV vs. IPVAW vs. SHW vs. STH) and a between-subjects factor relative to the number of bystanders in the scenario (Single bystander vs. Several bystanders). Consistent with this design, 5 × 2 mixed ANOVAS were performed with intra-subject (Type of violence; 5 levels) and inter-subject (Number of bystanders; 2 levels) factors for the dependent variables: perceived seriousness; responsibility attributed to the victim; responsibility attributed to the perpetrator; responsibility attributed to the bystander; and willingness to intervene by engaging in the different bystander’s behaviors. Partial eta squared was used as the estimator for the size effect. In the paired comparison, the *p*-values were adjusted by applying the Bonferroni correction.

These analyses were carried out with the SPSS 25 program.

### Results

#### Perceived seriousness and responsibilities attributed

The mixed ANOVA showed no significant effects of interaction between the independent variable of violence and the number of bystanders with regard to the dependent variables of perceived seriousness [*F*(2.804,316.867) = 0.913, *p* = 0.430), responsibility attributed to the victim [*F*(1.895,214.124) = 1.378, *p* = 0.254], responsibility attributed to the perpetrator [*F*(2.017,227.900) = 0.484, *p* = 0.618], and responsibility attributed to the bystander [*F*(3.504,395.981) = 0.621, *p* = 0.626].

The statistics corresponding to the analysis of the main effects for these two independent variables will now follow (Table 5).

**TABLE 5:** Main effects of type of violence and number of bystanders on perceived seriousness and responsibilities.

Factor	*M* (SE)	*F*	*d.f.*	*p*	η^2^
**Perceived seriousness**					
**Type of violence**					
RMV	2.97 (0.06)	50.169	2.804	<0.001	0.307
RFV	3.05 (0.06)				
IPVAW	3.61 (0.05)				
SHW	3.56 (0.05)				
STH	3.66 (0.05)				
**Number of bystanders**					
Single bystander^a^	3.39 (0.05)	0.291	1	0.591	
Several bystanders^b^	3.35 (0.05)				
**Victim’s responsibility**					
**Type of violence**					
RMV	1.89 (0.07)	99.201	1.895	<0.001	0.467
RFV	1.92 (0.07)				
IPVAW	1.35 (0.05)				
SHW	1.04 (0.02)				
STH	1.02 (0.01)				
**Number of bystanders**					
Single bystander^a^	1.39 (0.05)	2.544	1	0.114	
Several bystanders^b^	1.50 (0.05)				
**Perpetrator’s responsibility**					
**Type of violence**					
RMV	3.86 (0.05)	2.157	2.017	0.118	
RFV	3.85 (0.05)				
IPVAW	3.84 (0.04)				
SHW	3.93 (0.03)				
STH	3.94 (0.03)				
**Number of bystanders**					
Single bystander^a^	3.87 (0.04)	0.094	1	0.760	
Several bystanders^b^	3.89 (0.04)				
**Bystander’s responsibility**					
**Type of violence**					
RMV	2.42 (0.07)	55.068	3.504	<0.001	0.328
RFV	2.43 (0.07)				
IPVAW	2.91 (0.07)				
SHW	2.90 (0.07)				
STH	3.28 (0.06)				
**Number of bystanders**					
Single bystander^a^	2.87 (0.07)	2.657	1	0.106	
Several bystanders^b^	2.70 (0.08)				

RMV, robbery – male victim; RFV, robbery – female victim; IPVAW, intimate partner violence against women; SHW, sexual harassment at work; STH, street harassment. *M*, estimated mean; SE, standard error.

^a^*n* = 61.

^b^*n* = 54.

To begin, we can identify (Table 5) no main effects of the factor for number of bystanders on perceived seriousness (*p* = 0.591), the responsibility attributed to the victim (*p* = 0.114), to the perpetrator (*p* = 0.760), or to the bystander (*p* = 0.106). Nor can we identify main effects of the factor for type of violence on the responsibility attributed to the perpetrator (*p* = 0.118), although effects seen on the remainder of the dependent variables analyzed.

**FIGURE 1:**
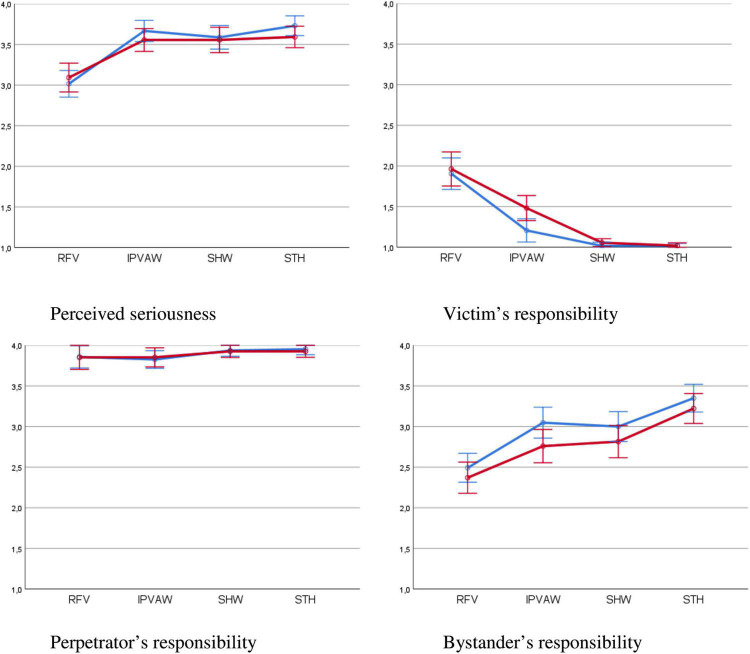
Perceived seriousness and responsibilities attributed by type of violence and number of bystanders (95% confidence intervals). RFV, robbery – female victim; IPVAW, intimate partner violence against women; SHW, sexual harassment at work; STH, street harassment. Blue line, single bystander; red line, several bystanders.

We must also point out that a paired comparison showed an absence of significant differences among the common violence case scenarios (RMV and RFV) (*p* = 1.0). In other words, in the common violence case analyzed (theft), the victim’s gender affects neither the perceived seriousness of the violence nor the degree to which responsibility is attributed for the incident or for eventual intervention. Therefore, according to this result and for greater clarity, all subsequent analyses compared the common violence scenario with a female victim (RFV) with the different forms of VAW, thus maintaining the gender of the victim constant in all scenarios. These comparisons are shown in Figure 1.

With regard to perceived seriousness, a significant (30.7%) effect size was observed with regard to type of violence (Table 5). Specifically, the comparisons made it possible to determine that the three cases of VAW (IPVAW, SHW, and STH) are perceived as equally serious (*p*-values between 0.979 and 1.0), but more serious than the common violence scenario RFV (*p* < 0.001) (see Figure 1).

Regarding responsibility attributed to the victim (Table 5), the type of violence introduces significant differences with a high (46.7%) effect size. The comparisons showed (Figure 1) that significantly less responsibility was attributed to the victim in the three scenarios of VAW than in the common violence scenario (RFV) (*p* < 0.001); and in the VAW scenario, less responsibility was attributed to the victim in the cases of SHW and STH than in the case of IPVAW (*p* < 0.001).

Finally, regarding the responsibility attributed to the bystander, the type of violence also introduced significant differences with a considerable (32.8%) size effect. The comparisons made (Figure 1) indicated that the participants attributed significantly more responsibility to the bystanders in cases of VAW than in those of common violence (RFV) (*p* < 0.001). Of the three forms of VAW analyzed, the STH scenario showed a significantly higher attribution of blame (*p* < 0.001), while no significant differences were seen between IPVAW and SHW (*p* = 1.0).

#### Positive or active bystander helping responses

No significant effects of interaction were detected between the type of violence and the number of bystanders for any of the positive or active bystander’s behaviors studied: “confront the perpetrator” [*F*(3.265,368.953) = 0.647, *p* = 0.598], “call the police / alert the authorities” [*F*(3.226,364.547) = 0.468, *p* = 0.719], “help the victim” [*F*(3.115,352.046) = 0.203, *p* = 0.900], and “ask other people for help” [*F*(3.374,381.269) = 1.323, *p* = 0.264].

Table 6 lists the statistics corresponding to the main effects of both independent variables.

**TABLE 6:** Main effects of type of violence and number of bystanders on positive or active bystander’ behaviors.

Factor	*M* (SE)	*F*	*d.f.*	*p*	η^2^
**Confront the perpetrator**					
**Type of violence**					
RMV	2.42 (0.07)	13.682	3.265	<0.001	0.108
RFV	2.56 (0.07)				
IPVAW	2.54 (0.08)				
SHW	2.80 (0.08)				
STH	2.92 (0.08)				
**Number of bystanders**					
Single bystander^a^	2.69 (0.08)	0.463	1	0.498	
Several bystanders^b^	2.61 (0.09)				
**Call the police/alert the authorities**					
**Type of violence**					
RMV	3.29 (0.08)	3.326	3.226	0.017	0.029
RFV	3.30 (0.07)				
IPVAW	3.51 (0.06)				
SHW	3.32 (0.08)				
STH	3.23 (0.08)				
**Number of bystanders**					
Single bystander^a^	3.35 (0.08)	0.109	1	0.742	
Several bystanders^b^	3.31 (0.08)				
**Help the victim**					
**Type of violence**					
RMV	3.45 (0.06)	11.519	3.115	<0.001	0.093
RFV	3.50 (0.06)				
IPVAW	3.60 (0.05)				
SHW	3.69 (0.05)				
STH	3.77 (0.04)				
**Number of bystanders**					
Single bystander^a^	3.65 (0.06)	1.225	1	0.271	
Several bystanders^b^	3.56 (0.06)				
**Ask other people for help**					
**Type of violence**					
RMV	3.10 (0.08)	2.462	3.374	0.055	
RFV	3.12 (0.08)				
IPVAW	3.26 (0.07)				
SHW	3.20 (0.07)				
STH	3.29 (0.07)				
**Number of bystanders**					
Single bystander^a^	3.31 (0.08)	4.092	1	0.045	0.035
Several bystanders^b^	3.07 (0.09)				

RMV, robbery – male victim; RFV, robbery – female victim; IPVAW, intimate partner violence against women; SHW, sexual harassment at work; STH, street harassment. *M*, estimated mean; SE, standard error.

^a^*n* = 61.

^b^*n* = 54.

**FIGURE 2:**
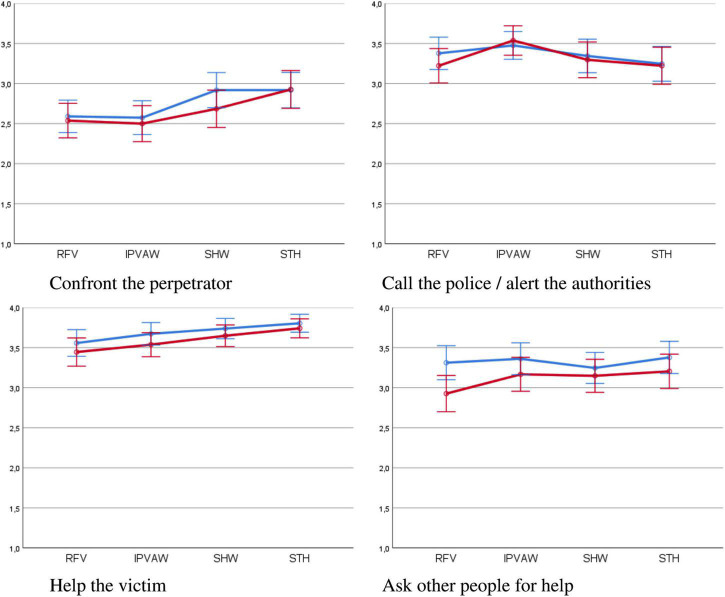
Positive or active bystander’ behaviors by type of violence and number of bystanders (95% confidence intervals). RFV, robbery – female victim; IPVAW, intimate partner violence against women; SHW, sexual harassment at work; STH, street harassment. Blue line, single bystander; red line, several bystanders.

Significant effects were detected within the remaining dependent variables for the type of violence (Table 6) and, once again, the paired comparison indicated an equivalent probability of positive response for both scenarios of common violence (RMV and RFV) (*p*-values between 0.111 and 1.0). Accordingly, subsequent comparisons only took into account the common violence scenario with a female victim (RFV), thus maintaining the gender of the victim constant in all scenarios (Figure 2).

The type of violence produced a significant effect on the probability of the option “confront the perpetrator,” with an effect size of 10.8% (Table 6). The likelihood of this response was greater in the case of STH, with significant differences with regard to RFV and IPVAW (*p* < 0.001); while the probability was smaller for IPVAW than for SHW (*p* = 0.002), and with no differences with regard to a RFV (*p* = 1.0).

For the response “call the police / alert the authorities” (Table 6), significant differences can be observed between the different types of violence with a medium-low size effect (2.9%). As can be seen in Figure 2, although the cases of VAW indicated response probabilities equivalent to RFV (IPVAW, *p* = 0.077; SHW and STH, *p* = 1.0), between these two options, a greater probability of notifying the authorities can be seen in the case of IPVAW than in the case of STH (*p* = 0.015).

Finally, the probability of help the victim is also significantly affected by the type of violence, with an effect size of 9.3%. No differences were detected between RFV and IPVAW (*p* = 1.0); however differences were found between the SHW and STH case scenarios (*p* = 0.004 and *p* < 0.001, respectively), for which the positive response was greater, with no differences between the two (*p* = 0.481). In turn, the probability of this response was significantly greater in the case of STH, compared to that of IPVAW (*p* = 0.039).

#### Negative or passive bystander responses

No significant effects of interaction were noted between the type of violence and the number of bystanders for any of the negative or passive bystander’s behaviors studied: “reproach the victim for her actions” [*F*(1.907,215.466) = 2.704, *p* = 0.072], “do not know what to do, would freeze up” [*F*(3.356,379.191) = 0.527, *p* = 0.684], “do nothing because it’s not my concern” [*F*(3.090,349.184) = 1.234, *p* = 0.297], “do nothing out of fear” [*F*(3.305,373.515) = 0.332, *p* = 0.821].

Table 7 lists the statistics corresponding to the main effects of both independent variables.

**TABLE 7:** Main effects of type of violence and number of bystanders on negative or passive bystander’ behaviors.

Factor	*M* (*SE*)	*F*	*d.f.*	*p*	η^2^
**Reproach the victim for her actions**					
**Type of violence**					
RMV	1.68 (0.07)	56.792	1.907	<0.001	0.334
RFV	1.64 (0.07)				
IPVAW	1.26 (0.05)				
SHW	1.02 (0.01)				
STH	1.02 (0.01)				
**Number of bystanders**					
Single bystander^a^	1.27 (0.05)	3.106	1	0.081	
Several bystanders^b^	1.38 (0.05)				
**Do not know what to do, would freeze up**					
**Type of violence**					
RMV	1.72 (0.06)	0.417	3.356	0.763	
RFV	1.67 (0.06)				
IPVAW	1.66 (0.06)				
SHW	1.64 (0.06)				
STH	1.69 (0.07)				
**Number of bystanders**					
Single bystander^a^	1.81 (0.06)	8.491	1	0.004	0.070
Several bystanders^b^	1.54 (0.07)				
**Do nothing because it is not my concern**					
**Type of violence**					
RMV	1.45 (0.06)	18.388	3.090	<0.001	0.140
RFV	1.41 (0.06)				
IPVAW	1.28 (0.04)				
SHW	1.21 (0.04)				
STH	1.08 (0.03)				
**Number of bystanders**					
Single bystander^a^	1.29 (0.05)	0.009	1	0.926	
Several bystanders^b^	1.28 (0.05)				
**Do nothing out of fear**					
**Type of violence**					
RMV	1.59 (0.06)	0.322	3.305	0.828	
RFV	1.58 (0.07)				
IPVAW	1.56 (0.06)				
SHW	1.53 (0.06)				
STH	1.57 (0.06)				
**Number of bystanders**					
Single bystander^a^	1.68 (0.07)	4.561	1	0.035	0.039
Several bystanders^b^	1.46 (0.08)				

RMV, robbery – male victim; RFV, robbery – female victim; IPVAW, intimate partner violence against women; SHW, sexual harassment at work; STH, street harassment. *M*, estimated mean; SE, standard error.

^a^*n* = 61.

^b^*n* = 54.

**FIGURE 3:**
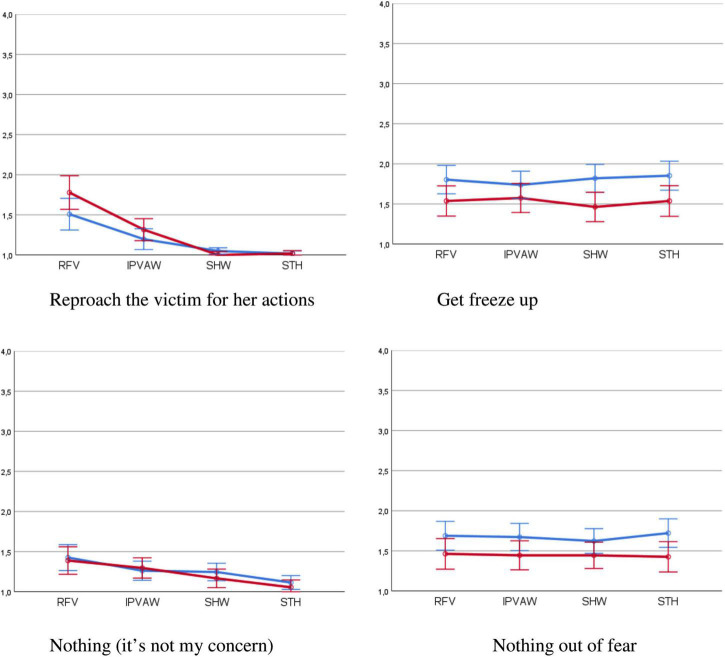
Negative or passive bystander bystander’s behaviors by type of violence and number of bystanders (95% confidence intervals). RFV, robbery – female victim; IPVAW, intimate partner violence against women; SHW, sexual harassment at work; STH, street harassment. Blue line, single bystander; red line, several bystanders.

No significant effects for the type of violence factor were found with respect to the likelihood of “do not know what to do, would freeze up” (*p* = 0.763) or for “do nothing out of fear” (*p* = 0.828). However, there are observable differences with regard to the number of bystanders, with medium-low size effects (7.0 and 3.9%, respectively). In both cases, the probability of these responses was greater when there was only one single bystander. In contrast, for the responses reproach the victim and “do nothing because it’s not my concern,” the number of bystanders had no effect (*p* = 0.081 and *p* = 0.926, respectively), although the type of violence did. As with the previous analyses, paired comparisons showed no significant differences for these responses between the two cases of common violence (RMV and RFV) (*p* = 1.0); consequently the only case scenario used was that in which the victim is a woman (RFV) (Figure 3).

Regarding the response reproach the victim (Table 7), a significant effect can be observed for the type of violence, with a considerable size effect (33.4%). The comparisons show that this response was less likely in any of the three cases of VAW (*p* < 0.001) than in the scenario of common violence (RFV); and among the VAW case scenarios, the probability was significantly less in the cases of SHW and STH than in those of IPVAW (*p* < 0.001) (Figure 3).

Finally, the probability of “do nothing because it’s not my concern” varied according to the type of violence, with a medium-high size effect (14%). Specifically, significant differences were found between common violence (RFV) and cases of SHW (*p* = 0.002) and STH (*p* < 0.001), which shows a lower negative response (Figure 3). On the other hand, no differences were found between common violence (RFV) and IPVAW (*p* = 0.179). Among the three forms of VAW analyzed, the scenarios of IPVAW and SHW showed an equivalent (*p* = 1.0) and greater probability of this response when compared to the STH scenario (*p* < 0.001 and *p* = 0.042, respectively).

### Discussion

The objective of this study was to test the usefulness of the QIHVC tool to explore the empirical relationship between perception and behavioral intention in the face of violent behavior, and other theoretically related variables (type of violence and number of bystanders present at the scene). The relationship between the scores obtained with a measuring tool and other external variables is one of the sources of information traditionally used in the process of validating measuring tools (Elosúa and Egaña, 2000). Its usefulness has been corroborated by different theoretical perspectives such as external focus of validity (Messick, 1989), the nomological validity focus (Campbell, 1960), and the nomothetic amplitude focus (Whitely, 1983). Each of these coincide generally in that the functionality of a tool is rooted in its ability to indicate empirical connections between the score and other theoretically related constructs. The standards of measurement already incorporate this aspect in their first versions, and remain in effect in the latest revision (American Educational Research Association [AERA] et al., 2014). The exploratory analysis of QIHVC in the pilot study provides results that confirm the empirical relationship between the scores obtained from the tool and the external variables with which, theoretically, this relationship is expected (i.e., type of violence and number of bystanders at the scene). Supported by these preliminary studies, the appropriateness and usefulness of the tool regarding the objective for which it was designed awaits new studies that may provide further empirical evidence.

The intrasubject comparisons confirm statistically significant differences for both the characterization of the types of violence and behavioral intent. With respect to the characterization of violence described in the case scenarios, differences are found in the degree of seriousness attributed to the violence, in the responsibility attributed to the victim, and in the responsibility attributed to the bystander in acting as an active agent. These results confirm that, under conditions of violence with similar intensity, the instrument is able to capture different levels of seriousness according to the type of violence. Likewise, the responsibility attributed to the victim varies according to the type of violence, as does the responsibility to intervene as a bystander. The lack of difference in responsibility attributed to the aggressor (ranging from 3.84 to 3.94) could indicate that, independently of the level of seriousness attributed to the incident, the origin of the aggression is clearly attributed to the aggressor in all scenarios described, which would not represent a challenge to the validity of the tool to compare different types of violence.

With regard to the intention of response, intrasubject comparisons also provide evidence as to the usefulness of the tool to compare the bystander’s willingness to engage in the face of different types of violence. Consequently, statistically significant differences can be observed in all of the active or positive answers: “confront the aggressor,” “call the police / alert the authorities,” and “help the victim.” However, the positive response “ask other people for help” varies as a function of the number of bystanders at the scene. With regard to passive or negative responses, significative differences can also be observed in responses such as “reproach the victim for her actions” and ignore the situation (“do nothing because it’s not my concern”). In the case “get freeze up” and “do anything out of fear,” the probability of response depends on the number of bystanders at the scene.

Ultimately, despite the small and homogeneous sample size of the third study, which constitute its primary limitation, the results obtained from this pilot sample can be said to confirm the usefulness of QIHVC to reach its intended purpose, which was to grasp the differences between the different types of violence, not just in their different characterization, but also in the behavioral intent of the bystander. Further in-depth studies with a broader and more heterogeneous sample size would be required to confirm these results and the adequacy of the QIHVC.

## General discussion

The main purpose of this study was to develop a tool that, by applying a contrastive methodology for its application in different forms of VAW (Katz et al., 2015; Levy and Ben-David, 2015; Rincón-Neira, 2017; Cinquegrana et al., 2018; Franklin and Garza, 2021; León et al., 2022), would allow measuring the probability of occurrence of bystander response in the face of these types of violence with good evidence of content validity. Specifically, an instrument was designed to apply the methodology of case scenarios to evaluate three forms of VAW (IPVAW, SHW, and STH): QIHVC.

To achieve this objective, the three case studies presented in this study were carried out: the development of a preliminary version of the QIHVC based on the theoretical conception of the three forms of VAW to be studied [defined by the Organic Laws 1/2004, 3/2007 and 10/2022, and by Bowman (1993), respectively] and the bystander responses analyzed in previous studies on this topic (e.g., Fundación FEDE. Social Research Service, 2012; León, 2020; León et al., 2022) (Study 1); a modified Delphi study (Lentz, 2007) to obtain other evidence of validity based on the content of the QIHVC (American Educational Research Association [AERA] et al., 2014) (Study 2); and a pilot study (Study 3) to determine the adequacy to capture differences in the characterization of the different forms of VAW studied compared to common violence and possible bystander’s behaviors in the face of these different forms of violence.

The main result of the three studies is the development of a set of case scenarios (see Supplementary Appendix 1) and a questionnaire related to its content (see Supplementary Appendix 2) which constitutes the QIHVC and, in its initial approximation, seems to constitute an adequate and sensible tool to capture the differences between the characterizations of common violence and VAW and in the possible response of bystanders in the face of such violence. It should be noted that, although previously developed tools based on a scenario methodology were available for use in the Spanish population (e.g., Rincón-Neira, 2017; León et al., 2022) they focused exclusively on IPVAW, while QIHVC includes two additional forms of VAW (SHW and STH) which allow for the establishment of comparisons with a common type of violence (RMV and RFV).

Nevertheless, this tool is not without limitations. For example, while Studies 1 and 2 gather the perspective of professionals and/or individuals with a high level of expertise in the field of VAW, it is necessary to delve further in obtaining empirical evidence to demonstrate the suitability of this tool for the intended objective.

Study 3 constitutes the first approach to this end, although, as previously noted, the limited sample size and its homogeneity (having been made up entirely of university students), could be considered its main limitation and it would require further analysis using wider and more heterogeneous sample studies before we can ascertain without any doubt the adequacy of this tool.

Moreover, Jouriles et al. (2016) have pointed out that despite certain advantages, self-report measures that aim to measure bystander responses are not without limitations. Thus, the importance of basing the responses on retrospective answers, as in the case of BBS, one of the most widely used self-report studies (Banyard et al., 2005, 2007, 2014; Banyard and Moynihan, 2011), and BBS-R (McMahon et al., 2014). However, the QIHVC, in line with the BAS (Banyard et al., 2005) and the BAS-R (McMahon et al., 2014), the BIB (Burn, 2009), or the WI-IPVAW (Gracia et al., 2018), aims to study the probability of intervention (while eliminates the need for retrospection). Moreover, as with the tools designed by Katz et al. (2015), Levy and Ben-David (2015), Papendick and Bohner (2017), Cinquegrana et al. (2018), Franklin and Garza (2021), or León (2020) and León et al. (2022) a scenario-based methodology was used to facilitate the understanding of what constitutes a situation of VAW. This does not ensure, however, as Jouriles et al. (2016) have noted, the absence of biases resulting from the understanding and interpretation of the situations described and the alternative bystander responses that are presented to the participants. To this we add the current context of social sensitivity in the face of VAW, which could easily lead to activate social desirability in the responses (Paulhus and Vazire, 2007; Gracia and Lila, 2015; Gracia et al., 2015; Ferrer-Pérez et al., 2019). It would be necessary to control this possible tainted factor in future studies in an attempt to minimize the risk of over-estimation in bystander response for more socially accepted answers, and the underestimation for those that would generate greater social rejection.

In any case, the greatest strength of QIHVC is that it offers an opportunity to analyze the bystander responses in the face of different forms of VAW compared to a form of common violence (RMV and RFV), which sets it apart from previous tools focused on specific forms of VAW such as sexual violence or IPVAW. Future lines of action include further work to determine the adequacy of the questionnaire designed. In particular, its application to wider and more heterogeneous sample sizes is critical for determining its proper functionality in general populations. Another interesting lines of work are a deeper analysis of the bystander responses included in the questionnaire grouping them to improve the analysis, or their adaptation to other populations such us adolescents. For this reason, it is necessary to continue studying whether in fact the eight response options included are the most appropriate or, whether it would be more adequate to group in some way.

## Data availability statement

The raw data supporting the conclusions of this article will be made available by the authors, without undue reservation.

## Ethics statement

The studies involving human participants were reviewed and approved by the Ethics Committee of University of the Balearic Islands (UIB 123CER19, 19-11-2020). The patients/participants provided their written informed consent to participate in this study.

## Author contributions

VF-P, AS-P, EB-F, and CD-A conceived and designed the research and the study 1. AN-R performed the study 2. LV-G performed the study 3. CD-A and AS-P analyzed the data. VF-P, AS-P, EB-F, CD-A, AN-R, and LV-G wrote the manuscript and approved the final manuscript. VF-P and AS-P critically revised the manuscript. All authors contributed to the article and approved the submitted version.
