# Supplementary material for: The Questionnaire of Intention to Help in VAW Cases (QIHVC): Development and preliminary results
Source: Front Psychol. 2023 Mar 22;14:1153678. doi: 10.3389/fpsyg.2023.1153678 (PMC10074592; doi:10.3389/fpsyg.2023.1153678)
Supplement: Supplementary file 1 [file Data_Sheet_1.docx]

Supplementary Material

The Questionnaire of Intention to Help in VAW Cases (QIHVC): Development and preliminary results

Victoria A. Ferrer-Perez*, Andrés Sánchez-Prada, Esperanza Bosch-Fiol, Carmen Delgado-Alvarez, Leila I. Vázquez-González, Ainara Nardi-Rodriguez

*** Correspondence:** Corresponding Author: victoria.ferrer@uib.es

# Supplementary Tables

*Appendix 1*. Case scenarios in study 1 and study 2

|  |  | Study 1 | Study 2 |
| --- | --- | --- | --- |
| RMV | Case scenario 1 | **ANTONIO** is having a few beers in a local bar near his home. Imagine that **you are sitting with a group of friends at the table next to his**. When ANTONIO decides to step outside for a smoke, he leaves his belongings on the table. A few minutes later **a man enters** the bar. All of a sudden ANTONIO, you and your friends witness the man **rushing out carrying his (Antonio’s) high end mobile** in his hand. | **ANTONIO** is having a few beers in a local bar near his home. Imagine that **you are sitting with a group of friends at the table next to his**. When ANTONIO decides to step outside for a smoke, he leaves his belongings on the table. A few minutes later **a man enters** the bar. All of a sudden ANTONIO, you and your friends witness the man **rushing out carrying his (Antonio’s) high end mobile** in his hand. |
|  | Case scenario 6 | **ANTONIO** is having a few beers in a local bar near his home. It’s his first time in the bar and he is surprised to find it so empty. **There is only one other table occupied (by you and a few others you’re having a drink with at the table next to his)**. At one point ANTONIO decides to step outside for a smoke, leaving his belongings on the table. A few minutes later ANTONIO sees **a man who enters** the bar and soon after **rushes out carrying his (Antonio’s) high end mobile** | **ANTONIO** is having a few beers in a local bar near his home. Imagine that **you are sitting with a group of friends at the table next to his**. When ANTONIO decides to step outside for a smoke, he leaves his belongings on the table. A few minutes later **a man enters** the bar. All of a sudden ANTONIO, you and your friends witness the man **rushing out carrying his (Antonio’s) high end mobile** in his hand. |
| RFV | Case scenario 2 | **MARIA** is having a few beers in a local bar near her home. It’s her first time in the bar and she is surprised to find it so empty. **There is only person sitting at the table next to hers (you)**. At one point MARIA decides to step outside for a smoke, leaving her belongings on the table. A few minutes later MARIA sees **a man who enters** the bar and soon after **rushes out carrying her high end mobile** | **MARIA** is having a few beers in a local bar near her home. Imagine that **you are sitting at the table next to hers and that (**besides MARIA) **you are the only other person in the bar**. When MARIA decides to step outside for a smoke, she leaves her belongings on the table. A few minutes later **a man enters** the bar. All of a sudden MARIA and you witness the man **rushing out carrying her**  **high end mobile** in his hand. |
|  | Case scenario 7 | **MARIA** is having a few beers in a local bar near her home. It’s her first time in the bar and she is surprised to find it so empty. **There is only one other table occupied (by you and a few others you’re having a drink with at the table next to hers)**. At one point MARIA decides to step outside for a smoke, leaving her belongings on the table. A few minutes later MARIA sees **a man who enters** the bar and soon after **rushes out carrying her high end mobile** | **MARIA** is having a few beers in a local bar near her home. Imagine that **you are sitting with a group of friends at the table next to hers**. When MARIA decides to step outside for a smoke, she leaves her belongings on the table. A few minutes later **a man enters** the bar. All of a sudden MARIA, you and your friends witness the man **rushing out carrying her high end mobile** in his hand. |
| IPVAW | Case scenario 3 | **LUCÍA and ALBERTO have been a couple** for 8 years and have been living together for 6. At the beginning of their relationship they had their differences, but things changed once they moved in together. When **ALBERTO** sees LUCÍA act in a way that displeases him, **he yells to the point of insulting her and has even pounded some pieces of furniture in their home**. LUCÍA feels a bit confused by this behavior, she’s afraid and feels bad, but thinks that despite their frequency, these are momentary outbursts due to something she has done wrong and should change.  Because of the shared courtyard in the building where LUCIA and ALBERTO live, **ALBERTO’s screams and insults can only be heard by the person who lives on the same floor (you alone)** | **LUCÍA and ALBERTO have been a couple** for 8 years and have been living together for 6. At the beginning of their relationship things were going well, despite some differences, but since they moved in together, things have taken a turn for the worse. When **ALBERTO** sees LUCÍA act in a way that displeases him, **he yells to the point of insulting her and has even pounded some pieces of furniture in their home**. LUCÍA feels a bit confused by this behavior, she’s afraid and feels bad, but thinks that despite their frequency, these are just momentary outbursts.  Due to the layout of the building where LUCIA and ALBERTO live, **ALBERTO’s screams and insults and LUCIA’s crying** can only be heard by the person who lives on the same floor (you alone**)**. |
|  | Case scenario 8 | **LUCÍA and ALBERTO have been a couple** for 8 years and have been living together for 6. At the beginning of their relationship they had their differences, but things changed once they moved in together. When **ALBERTO** sees LUCÍA act in a way that displeases him, **he yells to the point of insulting her and has even pounded some pieces of furniture in their home**. LUCÍA feels a bit confused by this behavior, she’s afraid and feels bad, but thinks that despite their frequency, these are momentary outbursts due to something she has done wrong and should change.  Because of the shared courtyard in the building where LUCIA and ALBERTO live, **ALBERTO’s screams and insults can only be heard by the people who live on the same floor (you, among others)** | **LUCÍA and ALBERTO have been a couple** for 8 years and have been living together for 6. At the beginning of their relationship things were going well, despite some differences, but since they moved in together, things have taken a turn for the worse. When **ALBERTO** sees LUCÍA act in a way that displeases him, **he yells to the point of insulting her and has even pounded some pieces of furniture in their home**. LUCÍA feels a bit confused by this behavior, she’s afraid and feels bad, but thinks that despite their frequency, these are just momentary outbursts.  Due to the layout of the building where LUCIA and ALBERTO live, **ALBERTO’s screams and insults and LUCIA’s crying** can only be heard by the people who live on the same floor (you, among others) |
| SHW | Case scenario 4 | **RAQUEL** recently started to work as a **secretary** at a company. RAQUEL **shares an office with another person (you)**. On several occasions, you have observed how **JUAN, the company director**, **constantly looks** at RAQUEL and **refers to her as “babe”**, and have noticed that RAQUEL is clearly uncomfortable in this situation. One day, **while you and RAQUEL are working**, JUAN enters the office to explain to RAQUEL some administrative procedures she must perform and, while he speaks, **he gets very close to her and places his hand on her leg**. She becomes very nervous and moves away abruptly and looks to you | **RAQUEL** recently started to work as a **secretary** at a company. RAQUEL is young and tends to dress very nicely for work. On several occasions, you have observed how **JUAN, the company director**, **constantly looks** at RAQUEL and **refers to her as “babe”**, and have noticed that RAQUEL is clearly uncomfortable in this situation. One day, **while you and RAQUEL are working**, JUAN enters the office to explain to RAQUEL some administrative procedures she must perform; while he speaks, **he gets very close to her and places his hand on her thigh**. RAQUEL becomes very nervous and moves away abruptly |
|  | Case scenario 9 | **RAQUEL** recently started to work as a **secretary** at a company. RAQUEL **shares an office with several people (you and a few others)**. On several occasions, the other people in the office have observed how **JUAN, the company director**, **constantly looks** at RAQUEL and **refers to her as “babe”**, and have noticed that RAQUEL is clearly uncomfortable in this situation. One day, **while RAQUEL and the others in the shared office are working**, JUAN enters the office to explain to RAQUEL some administrative procedures she must perform and, while he speaks, **he gets very close to her and places his hand on her leg**. She becomes very nervous and moves away abruptly and looks to you and the others | **RAQUEL** recently started to work as a **secretary** at a company. RAQUEL is young and tends to dress very nicely for work. On several occasions, you and the other people who share a workspace have observed how **JUAN, the boss**, **constantly looks** at RAQUEL and **refers to her as “babe”**, and have noticed that RAQUEL is clearly uncomfortable in this situation. One day, **while RAQUEL, you and the others in the shared workspace are working**, JUAN enters the office to explain to RAQUEL some administrative procedures she must perform; while he speaks, **he gets very close to her and places his hand on her thigh**. RAQUEL becomes very nervous and moves away abruptly |
| STH | Case scenario 5 | **CHLOE** decides to go to a party with some girlfriends. Before heading out, she puts on her favorite black party dress and some heels. When she arrives at the party, she has a couple of drinks, dances with some girlfriends and has a good time. She decides to return home alone since it's only a 10 minute walk.  On her way home, **she passes by a man** who **declares in a very loud voice that she is very pretty with her dress, but would look even better without it, and insists on walking her home**. CHLOE picks up the pace and even takes off her heels to run. **Only one person (you) is observing this incident from across the street** | **ANA** decides to go to a party with some girlfriends**.** Before heading out, she puts on her favorite black party dress and some heels. When she arrives at the party, she has a couple of drinks, dances with some girlfriends and has a good time. She decides to return home alone since it's only a 10 minute walk.  On her way home, **she passes by a stranger** who **declares in a very loud voice that she is very pretty with her dress, but would look even better without it, and insists on walking her home**. ANA is afraid, picks up the pace and tries to get away. **Only one person (you) is walking down the street at that moment and observes the scene** |
|  | Case scenario 10 | **CHLOE** decides to go to a party with some girlfriends. Before heading out, she puts on her favorite black party dress and some heels. When she arrives at the party, she has a couple of drinks, dances with some girlfriends and has a good time. She decides to return home alone since it's only a 10 minute walk.  On her way home, **she passes by a man** who **declares in a very loud voice that she is very pretty with her dress, but would look even better without it, and insists on walking her home**. CHLOE picks up the pace and even takes off her heels to run. **Only a few people (yourself included) observe this incident from across the street)** | **ANA** decides to go to a party with some girlfriends. Before heading out, she puts on her favorite black party dress and some heels. When she arrives at the party, she has a couple of drinks, dances with some girlfriends and has a good time. She decides to return home alone since it's only a 10 minute walk.  On her way home, **she passes by a stranger** who **declares in a very loud voice that she is very pretty with her dress, but would look even better without it, and insists on walking her home**. ANA is afraid, picks up the pace and tries to get away. **Only a few people (yourself included) are walking down the street at that moment and observe the scene** |

RMV: Robbery – Male victim; RFV: Robbery – Female victim; IPVAW: Intimate Partner Violence against Women); SHW: Sexual Harassment at Work; STH: Street Harassment

*Appendix 2*. Questionnaire for each of the case scenarios in study 1 and study 2

| Study 1 | Study 2 |
| --- | --- |
| How would you rate the seriousness of this case? | How would you rate the seriousness of what has happened to NAME OF VICTIM? |
| To what extent do you believe that NAME OF THE VICTIM provoked the situation? | To what extent do you believe that NAME OF VICTIM is responsible for this situation? |
| To what extent do you believe that THE PERPETRATOR is responsible for the situation? | To what extent do you believe that THE PERPETRATOR is responsible for this situation? |
| To what extent would you consider yourself responsible for intervening if you were witness to such an incident? | To what extent would you consider yourself responsible for intervening (do something) if you were witness to this situation? |
| If you were witness to this incident, what is the probability that you would take the following actions | If you were witness to this incident, what is the probability that you would take the following actions: |
| I would confront NAME OF VICTIM | Reproach NAME OF VICTIM for her actions |
| I would confront the PERPETRATOR | I would confront the PERPETRATOR |
| I would call the police and I would confront the PERPETRATOR | I would call the police / alert the authorities |
| I would call the police, but NOT confront the PERPETRATOR |  |
| I would try to mediate, if possible, between NAME OF VICTIM and the PERPETRATOR | --------- |
| I would try to help NAME OF VICTIM | I would try to help NAME OF VICTIM |
| I would ask … for help  other people (Scenario 1)  the other bystanders (Scenario 2) | I would ask other people for help |
| I wouldn’t know what to do | I wouldn’t know what to do, I would freeze up |
| I would probably do nothing | -------- |
| I would do nothing (it’s not my concern) | I would do nothing because it’s not my concern |
|  | I would do nothing out of fear |
